# Perceived Discrimination among Black Youth: An 18-Year Longitudinal Study

**DOI:** 10.3390/bs8050044

**Published:** 2018-04-27

**Authors:** Shervin Assari, Frederick X. Gibbons, Ronald L. Simons

**Affiliations:** 1 Department of Psychiatry, University of Michigan, 4250 Plymouth Road, SPC 5763, Ann Arbor, MI 48109-2700, USA; 2 Center for Research on Ethnicity, Culture and Health, School of Public Health, University of Michigan, Ann Arbor, MI 48109-2700, USA; 3 Department of Psychological Sciences, University of Connecticut, Storrs, CT 06269, USA; rick.gibbons@uconn.edu; 4 Department of Sociology, University of Georgia, Athens, GA 30602, USA; rsimons@uga.edu

**Keywords:** Blacks, African Americans, socioeconomic status (SES), education, discrimination, racism, place

## Abstract

**Background**: Recent research has suggested vulnerability to perceived racial discrimination (PRD) as a mechanism behind high levels of depression seen in high socioeconomic status (SES) Black males. To better understand the effects of gender and SES on shaping experiences of PRD among Black youth in the United States, we used data from the Family and Community Health Study (FACHS) to explore the trajectory of PRD in Black youth by gender, SES, and place. **Methods**: Data came from FACHS, 1997–2017, which followed 889 children aged 10–12 years old at Wave 1 (*n* = 478; 53.8% females and *n* = 411; 46.2% males) for up to 18 years. Data were collected in seven waves. The main predictors of interest were gender, SES (parent education and annual family income), age, and place of residence. Main outcomes of interest were baseline and slope of PRD. Latent growth curve modeling (LGCM) was used for data analysis. **Results**: Gender, SES, place, and age were correlated with baseline and change in PRD over time. Male, high family income, and younger Black youth reported lower PRD at baseline but a larger increase in PRD over time. Youth who lived in Iowa (in a predominantly White area) reported higher PRD at baseline and also an increase in PRD over time. High parental education was not associated with baseline or change in PRD. **Conclusion**: In the United States, Black youth who are male, high income, and live in predominantly White areas experience an increase in PRD over time. Future research is needed on the interactions between gender, SES, and place on exposure and vulnerability of Black youth to PRD. Such research may explain the increased risk of depression in high SES Black males.

## 1. Background

Perceived racial discrimination (PRD) is a risk factor for a wide range of undesired health outcomes across populations, particularly racial and ethnic minorities [1,2,3]. Among Black youth, PRD increases risk of mental health problems such as psychological distress [4,5], suicidal ideation [6,7] as well as psychiatric disorders [8] such as anxiety [9] and depression [10].

Recent literature has documented high levels of depression among high SES Black males across age groups including youth [11] and adults [12,13]. As male gender [14] and high SES [15] generally tend to be protective against the risk of depression, researchers have shown interest in understanding the mechanisms by which male gender and high SES become vulnerability factors for Blacks [16]. One explanation involves PRD: among Blacks and possibly other minority groups, males and high SES individuals report higher levels of PRD, and show stronger effects of PRD on their psychological well-being [11,13,16,17]. Although some support exists for this hypothesis [11,13,16,17], more research is needed on the role of gender and SES as vulnerability factors for experiencing PRD among Black youth.

Among minorities, including Blacks, males seem to be more vulnerable to the mental health effects of discrimination [14,18,19]. In a six- [20] and 12- [21] year follow up study of Black youth in Flint MI, an increase in PRD predicted worsening of anxiety and depression symptoms for male, but not female, Black youth. In another study, PRD better predicted substance use in Black males compared to Black females [18]. In another study on Blacks, recent PRD was a risk factor for smoking among Black males but among not Black females [19]. These patterns seem to not be specific to Blacks, as similar gender differences have been found in Arab Americans [4] and Hispanics [19].

In addition to male gender, high SES appears to be a vulnerability factor for mental health effects of PRD. Blacks, particularly Black males, show less health gain from each unit increase in SES [20]. PRD has been proposed as a mechanism for such “*diminished gain of SES*” for Blacks [20,21,22]. In this theory, Blacks gain less health benefits from SES because high SES Blacks experience more PRD than low SES Blacks. Again, there is some empirical evidence that supports this hypothesis [16,23]. In a study of Black men, high SES was associated with more PRD [16]. In another study, discrimination had a stronger effect on risk of depression in Black youth with higher subjective SES compared to their low SES counterparts [23].

As a result of high PRD among high SES Blacks, economic resources such as education [24], employment [25], social contacts [26], and neighborhood quality [27] show smaller effects on life expectancy for Blacks, compared to Whites [24,25]. Education had a stronger effect on pattern of alcohol use in Whites than Blacks [28]. Even self-efficacy [29] and perception of control over life [30] fail to be as protective for Blacks as they are for Whites.

### Aim

To better understand how gender, SES, and place influence the experience of PRD among Black youth in the United States, we used data from the Family and Community Health Study (FACHS) to explore baseline and trajectory of PRD among Black children and youth as a function of gender, SES, age, and place of residence. FACHS, the longest and largest cohort of Black families in the United States [31], provides a unique opportunity to study within-race heterogeneities in pattern of changes in PRD over time among sub-groups of Black youth. In line with the existing literature on higher exposure and vulnerability of males [4,18,19,21,32] and individuals with high SES [11,13,17] to PRD, we expected an increase in PRD among Black youth who were male, high SES, and were living in predominantly White areas.

## 2. Methods

### 2.1. Design and Setting

FACHS is an ongoing flagship longitudinal study of Black families in the U.S. The study recruited half of its sample from Iowa and half from Georgia, with an overall sample of 889 families at Wave 1 (year 1997) [33].

### 2.2. Ethics

The study protocol received approval from the Institutional Review Boards (IRBs) at Iowa State where FACHS started. The study protocol was also approved by the IRB at Dartmouth College, Iowa State University, University of Iowa, and University of Georgia. Written assent was received from all participants through age 18 (Wave 4). Informed consent was received from youth above age 18 and also their parents or caregivers who participated in the study. Participants received financial compensation for their time and participation.

### 2.3. Participants

Inclusion criteria for this study were families with an adolescent who was in 5th grade at Wave 1 and who self-identified as Black/African American race; and participation of a parent or a primary caregiver. Average age of the children at the time of enrollment to the study was 10.5 years. In 84% of the families, the parent was biological mother of the adolescent.

### 2.4. Sampling and Recruitment

Families were recruited from small metropolitan areas, rural communities, and suburbs in Iowa and Georgia, which were composed of lower and middle class families. Median annual family income at the time of enrolment was $20,803/year, and one third of families were below the poverty line. More detailed information regarding the FACHS sample and recruitment is available elsewhere [34,35].

### 2.5. Data Collection

Of the total families that were contacted, 72% provided data. Most common cause of declining was the amount of time the interviews took. All the interviews were done by Black/African American interviewers. All interviewers received extensive training. Each interview took three hours on average. (Data collection required two visits). Mode of interview was computer-assisted personal interview (CAPI), which increases quality of data when the questionnaire structure is complex [36]. Data were collected in Wave 1 (1997–1998), Wave 2 (1999–2000), Wave 3 (2002–2003), Wave 4 (2005–2006), Wave 5 (2007–2009), Wave 6 (2010–2011), and Wave 7 (2014–2016). Retention (of the original sample) was 79% through Wave 6 and then 61% at Wave 7.

### 2.6. Measures

*Sociodemographic characteristics*. The following sociodemographic variables were included: age at Wave 1 (operationalized as a continuous measure ranging from 9 to 13), gender (male versus female (referent category)), place (Iowa versus Georgia (referent category)), and parent SES (annual family income and parent education), and PRD. Family income and education (highest education attainment of parents) were both treated as continuous measures.

*Perceived Racial Discrimination (PRD)*. Participants reported their experience of racist events, using the 13-item version of the Schedule of Racist Events [37]. After describing various discriminatory events, participants were asked how often they have experienced each of the events. Example item includes “How often has someone said something insulting to you just because you are African American?” Item responses ranged from 1 (never) to 4 (several times) (αs = 0.86–0.90). PD is shown to predict poor health using this measure [33,38,39].

### 2.7. Statistical Analysis

IBM SPSS 22 (IBM Inc., Armonk, NY, USA) was used to perform our univariate and bivariate analyses. We used AMOS [40] to run Latent Growth Curve Modeling (LGCM). LGCM is a particular type of structural equation modeling (SEM) that allows modeling of linear and non-linear growth over time [41]. LGCM offers several advantages over traditional methods for longitudinal analyses. AMOS uses full information maximum likelihood (FIML) to accommodate missing data. To describe the sample, we used frequency table, mean, and standard deviation (SD). For bivariate analysis, Pearson correlation test was used.

First, we ran an unconditional LGCM that suggested our intercept and slope are significantly different from zero (they have a variance that can be modeled as outcomes). We ran models with and without quadratic slope (non-linear slope). As the fit did not change, and none of the paths from our predictors to quadratic slope were significant, we dropped the quadratic slope for our final model. We also compared models with and without correlated error between PRD observations. The fit did not improve, so we kept the most parsimonious model (without correlated errors).

In the next step, we estimated our conditional LGCMs, with gender, SES (parent education and family income), age, and place as predictors, and baseline and (linear) slope of PRD as outcomes. Paths were drawn from all predictors to the intercept as well as the linear slope.

We evaluated the model using conventional fit statistics including a comparative fit index (CFI) more than 0.90, a root mean squared error of approximation (RMSEA) of less than 0.08, and a Chi square to degrees of freedom ratio (CMIN/DF) of less than 4.0 [42,43,44,45,46]. We reported the standardized regression coefficient, the associated SE, and the p value for each path.

## 3. Results

### 3.1. Descriptive Statistics

Table 1 shows the descriptive statistics in the sample at baseline. Average age of the participants was 10 years old, with a range of nine to 13 at baseline. PRD increased over time from wave 1 and 2 to waves 3 and 4 and then declined at waves 5 to 7, as PRD was lower than its baseline at the final wave.

### 3.2. Bivariate Correlations

Table 2 summarizes the results of bivariate correlations between all study variables among the participants. Gender was not correlated with any of the study constructs including age, parent education, family income, and discrimination. Living in Iowa, however, was positively associated with education and both baseline and average PRD. Older age at baseline was associated with higher baseline PRD. Parent education and family income were negatively correlated with PRD at baseline.

### 3.3. Latent Growth Curve Model

Table 3 summarizes the path coefficients for the LGCM. The LGCM showed acceptable fit to the data (Chi-square = 178.148, Probability level = 0.000, CFI = 0.917, CMIN/DF = 4.049, df = 44, RMSEA = 0.059 (0.050–0.068)).

**Figure 1:**
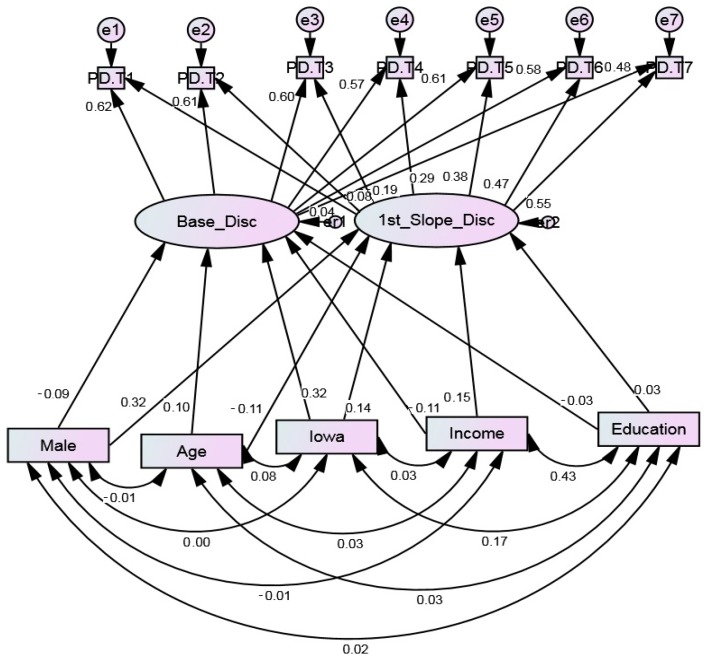
Summary of paths in the latent growth curve model on predictors of perceived discrimination over 18 years (*n* = 889). Chi-square = 178.148, Probability level = 0.000, CFI 0.917, CMIN/DF 4.049, df = 44, RMSEA = 0.059 (0.050–0.068).

This model shows that gender, family income, place, and age were correlated with baseline and change in PRD. Male, high income, and younger Black youth reported lower PRD at baseline but a larger increase in PRD over time. Children who lived in Iowa (a predominantly White area) reported higher PRD at baseline and also an increase over time. High parental education was not associated with baseline or change in PRD. (Table 3 and Figure 1)

## 4. Discussion

The current study shows that gender, place, and SES are among the main social determinants of PRD experience over an extended period of time among Black youth. Male gender, high SES (family income), and living in Iowa (a predominantly White community) are associated with an increase in experiencing PRD. Thus, high SES is not all protective for Blacks, as it may in fact increase Black youth’s exposure to PRD. Thus, gender, SES, and place operate as vulnerability factors that shape social patterning of PRD for Black youth.

It is still unknown whether PRD is the underlying mechanism behind high prevalence of depression in high SES Blacks [11,13] and stronger effect of PRD on depression and substance use among male [18,19,47,48] and high SES [16,23] Blacks. Although high SES is associated with both PRD [49] and depression [11,13], a recent study failed to find evidence for PRD as a mediator of the link between SES and depression in Black men [50]. Future research should test if high PRD mediates the positive association between SES and depression for Black men [5,51].

We explain our gender finding using some related theoretical frameworks. Subordinate Male Target Hypothesis [52,53] and the Outgroup Male Target Hypothesis [54,55] suggest that White males fiercely sustain their hierarchy to handicap Black males (and other men of color) more than Black females. The Male Warrior Hypothesis [54] implies that due to evolution, men’s’ mindsets and psychology are designed to facilitate intergroup success, which comes with increased intergroup conflict as a side effect. These hypotheses suggest that exposure to PRD is not merely due to race, but is shaped at the nexus of race and gender [55]. The notion that minority men are the primary target of intergroup negativity [55] advocates for the use of an intersectionality approach to study health in racial minorities [56,57].

More is known about how affluence confers risk for White [58,59] than Black [23,60] youth. For White youth, suburban, affluent communities may increase engagement in substance use, internalizing problems, and delinquency [58,59,61,62]. In predominantly White samples, neighborhood income may also be positively associated with youth delinquent behaviors and internalizing symptoms [63,64,65,66,67,68,69]. This line of research, although in Whites, suggest that youth risk derives from both ends of the economic spectrum [70,71].

The processes for increased risk of psychopathology and problem behaviors among affluent Black youth are, however, very different from those of White youth. Affluent male Black youth experience more racism and discrimination [49]. There is also some research suggesting that high SES Black youth are more vulnerable to the effects of PRD on depression [16,23]. Another explanation may be that living in predominantly White communities increases high SES Black families’ exposure to discrimination. This hypothesis was supported by the current finding that Black youth in Iowa reported more PRD than Black youth in Georgia.

Under the current social structure and system, society treats individuals based on their group membership and skin color. In the presence of structural racism and widespread discrimination, high SES may become a risk factor for Black youth. This phenomenon is in contrast with the mainstream work on social determinants of health [72,73,74]. Fundamental Cause Theory (FCT), for example, conceptualizes high SES as a protective rather than risk factor [72,73,74,75]. We suggest, however, that this is not the case for all groups under all circumstances. Under racism and in the presence of discrimination, high SES may come with hidden costs for minorities such as Blacks. The finding that Blacks who lived in predominantly White communities reported more PRD supports this hypothesis.

### 4.1. Limitations

Findings of the current study should be interpreted with regard to the study limitations. First of all, we could only control for a few confounders. Several important variables such as family type, attribution of discrimination, vigilance, racial identity, social support, coping, and other types of stress were not included. Second, we did not study the mechanism for the effects of gender, age, place, and SES and PRD. A wide range of social, cognitive, psychological, and behavioral processes may interfere with experience, expectation, and attribution of discrimination [10]. Despite these limitations, high sample size and long duration of follow up were among the strengths of this study.

### 4.2. Future Research

There is a need to study the interactive effects of race, ethnicity, gender, SES, and place of residence on PRD in causing racial health disparities. There is a need to study the role of other affective reactions such as anxiety and anger in explaining the effects of PRD [76,77]. The effects of the above social constructs are multiplicative rather than additive. Research should also test whether the same intersections influence the link between PRD and undesired physical and mental health outcomes. Such research will help us understand why male gender and high SES operate as vulnerability factors for PRD. One potential explanation is that males and high SES people do not expect discrimination or they find discrimination more unfair [78]. Another explanation is males and high SES Blacks may not have the same access to social support that can buffer the effects of PRD or may not use their social relations to discuss PRD. It is unknown whether male and high SES Blacks are more vigilant for discriminatory cues or are simply more discriminated against [79]. Future research should examine how resilience, coping, social support, individual behaviors, norms, and context explain differential exposure and vulnerability to PRD. Masculine ideologies and other social identities may have a role [80,81]. Mediational analysis is also suggested for future studies. Finally, it is unclear why objective SES indicators such as family income and education operate differently from subjective SES in increasing exposure and vulnerability to PRD [23].

### 4.3. Conclusions

To conclude, the findings reported here introduce male gender, high SES (family income), and living in predominantly White communities as vulnerability factors for PRD for Black youth. Future research should test whether PRD has a stronger link with psychopathology in males and high SES individuals than in females and those in low SES. Such research may explain the high prevalence of depression in high SES Blacks, and diminished effects of SES on the health of Blacks.

## Figures and Tables

**Table 1:** Descriptive statistics in the pooled sample (*n* = 889).

	*n*	Minimum–Maximum	Mean (SD)
Age (Years)	837	9.00–13.00	10.36 (0.71)
Parent Education ^a^ (1–7)	806	1.00–7.00	3.23 (1.13)
Annual Family Income ^a^ (USD)	784	0.00–221,100.00	28,471.19 (25,616.13)
Perceived racial discrimination (PRD, Wave 1)	824	1.00–3.62	1.63 (0.53)
Perceived racial discrimination (PRD, Wave 2)	732	1.00–3.77	1.63 (0.56)
Perceived racial discrimination (PRD, Wave 3)	715	1.00–3.77	1.72 (0.57)
Perceived racial discrimination (PRD, Wave 4)	714	1.00–3.77	1.77 (0.60)
Perceived racial discrimination (PRD, Wave 5)	689	1.00–3.69	1.65 (0.55)
Perceived racial discrimination (PRD, Wave 6)	698	1.00–4.00	1.55 (0.56)
Perceived racial discrimination (PRD, Wave 7)	541	1.00–4.00	1.46 (0.64)

Source: Family and Community Health Study (FACHS). ^a^ Reported by the primary caregiver, almost always mother of the participating youth.

**Table 2:** Correlation matrix of the study variables (*n* = 889).

	1	2	3	4	5	6	7
1 Gender (Women)	1	0.01	−0.01	0.02	−0.01	−0.05	0.05
2 Place of Residence (Iowa)		1	0.08 *	0.18 **	0.03	0.18 **	0.31 **
3 Age (Years)			1	0.03	0.03	0.10 **	0.06 ^#^
4 Parent Education ^a^ (1–7)				1	0.43 **	−0.07 *	0.01
5 Annual Family Income ^a^ (USD)					1	−0.15 **	−0.05
6 Baseline Perceived Racial Discrimination (PRD)						1	0.62 **
7 Mean Perceived Racial Discrimination (PRD)							1

^#^*p* < 0.1, * *p* < 0.05, ** *p* < 0.01. Source: Family and Community Health Study (FACHS). ^a^ Reported by the primary caregiver, almost always mother of the participating youth.

**Table 3:** Summary of paths in the latent growth curve model on predictors of perceived discrimination over 18 years (*n* = 889).

	B (SE)	*p*
**→ Baseline Perceived Racial Discrimination (PRD)**		
Gender (male)	−0.09 (0.03)	0.046
Place (Iowa)	0.32 (0.03)	<0.001
Age (Year)	0.10 (0.02)	0.029
Parent Education ^a^ (1–7)	−0.03 (0.02)	0.589
Annual Family Income ^a^ (USD1000)	−0.11 (0.00)	0.039
**→ Slope of Perceived Racial Discrimination (PRD)**		
Gender (male)	0.32 (0.00)	<0.001
Place (Iowa)	0.14 (0.00)	0.033
Age (Year)	−0.11 (0.00)	0.089
Parent Education ^a^	0.03 (0.00)	0.718
Annual Family Income ^a^ (USD1000)	0.15 (0.00)	0.050

Source: Family and Community Health Study (FACHS). B: Standardized path coefficient. ^a^ Reported by the primary caregiver, almost always mother of the participating youth.
